# Machine learning classification models for fetal skeletal development performance prediction using maternal bone metabolic proteins in goats

**DOI:** 10.7717/peerj.7840

**Published:** 2019-10-18

**Authors:** Yong Liu, Cristian R. Munteanu, Qiongxian Yan, Nieves Pedreira, Jinhe Kang, Shaoxun Tang, Chuanshe Zhou, Zhixiong He, Zhiliang Tan

**Affiliations:** 1 CAS Key Laboratory for Agro-Ecological Processes in Subtropical Region, Institute of Subtropical Agriculture, Chinese Academy of Sciences, Changsha, Hunan, China; 2 RNASA-IMEDIR, Computer Science Faculty, University of A Coruna, A Coruña, Spain; 3 Biomedical Research Institute of A Coruña (INIBIC), University Hospital Complex of A Coruña (CHUAC), A Coruña, Spain

**Keywords:** Fetal bone metabolism, Maternal malnutrition, Intrauterine growth retardation, Computational analysis, Machine learning

## Abstract

**Background:**

In developing countries, maternal undernutrition is the major intrauterine environmental factor contributing to fetal development and adverse pregnancy outcomes. Maternal nutrition restriction (MNR) in gestation has proven to impact overall growth, bone development, and proliferation and metabolism of mesenchymal stem cells in offspring. However, the efficient method for elucidation of fetal bone development performance through maternal bone metabolic biochemical markers remains elusive.

**Methods:**

We adapted goats to elucidate fetal bone development state with maternal serum bone metabolic proteins under malnutrition conditions in mid- and late-gestation stages. We used the experimental data to create 72 datasets by mixing different input features such as one-hot encoding of experimental conditions, metabolic original data, experimental-centered features and experimental condition probabilities. Seven Machine Learning methods have been used to predict six fetal bone parameters (weight, length, and diameter of femur/humerus).

**Results:**

The results indicated that MNR influences fetal bone development (femur and humerus) and fetal bone metabolic protein levels (C-terminal telopeptides of collagen I, CTx, in middle-gestation and N-terminal telopeptides of collagen I, NTx, in late-gestation), and maternal bone metabolites (low bone alkaline phosphatase, BALP, in middle-gestation and high BALP in late-gestation). The results show the importance of experimental conditions (ECs) encoding by mixing the information with the serum metabolic data. The best classification models obtained for femur weight (Fw) and length (FI), and humerus weight (Hw) are Support Vector Machines classifiers with the leave-one-out cross-validation accuracy of 1. The rest of the accuracies are 0.98, 0.946 and 0.696 for the diameter of femur (Fd), diameter and length of humerus (Hd, Hl), respectively. With the feature importance analysis, the moving averages mixed ECs are generally more important for the majority of the models. The moving average of parathyroid hormone (PTH) within nutritional conditions (MA-PTH-experim) is important for Fd, Hd and Hl prediction models but its removal for enhancing the Fw, Fl and Hw model performance. Further, using one feature models, it is possible to obtain even more accurate models compared with the feature importance analysis models. In conclusion, the machine learning is an efficient method to confirm the important role of PTH and BALP mixed with nutritional conditions for fetal bone growth performance of goats. All the Python scripts including results and comments are available into an open repository at https://gitlab.com/muntisa/goat-bones-machine-learning.

## Introduction

In the early stages of embryonic development of mammals, the fetal skeleton development is composed of fibrous membranes and hyaline cartilage. The bone is usually formed through endochondral ossification and intramembranous ossification regulated by intra- or extra-factors in the middle or late gestation. Chondroblasts play an extremely important role in chondrogenesis by forming chondrocytes and extracellular matrix (EM). Moreover, the mineral metabolism of fetus skeletal development is essentially dependent on parathyroid hormone (PTH), and PTH-related proteins (PTHrP) (Mendes et al., 2019), but not calcitonin, vitamin D/calcitriol, fibroblast growth factor (FGF-23) or sex steroids (Kovacs, 2015). For PTH, it is critical to regulate calcium and skeletal homeostasis, and fetal-placental mineral homeostasis (Simmonds et al., 2010), via calciotropic and phosphotropic hormones (Kovacs, 2014).

For bone formation, the new bone is formed by osteoblasts, and some biomarkers reflect the activity of osteoblasts. The homeostasis of bone formation and bone resorption is achieved and regulated through the local mediators and systemic hormones. In general, the most commonly measured bone formation biomarkers are the bone alkaline phosphatase (BALP) and its isoforms, osteocalcin (OC) (Lee et al., 2007), and the procollagen-breakdown products (procollagen type 1 N-terminal and C-terminal propeptides, PICP and PINP). The BALP is a bone-specific isoform of liver alkaline phosphatase (ALP) on the surface of osteoblasts, reflects the biosynthetic activity of bone-forming cells, secreted by the liver, bone, placenta or intestines (Shipman, Holt & Gama, 2013; Tanaka et al., 1997). Osteocalcin is a bone-derived hormone which affects glucose metabolism by regulating insulin secretion and sensitivity (Ferron & Lacombe, 2014). The quality of bone strength is characterized by the EM, turnover rate, and mineral homeostasis. EM contains hyaluronic acid, proteoglycans, glycoproteins and collagen (type I collagen, the most abundant protein secreted by osteoblasts in the procollagen form of PICP and PINP) (Wallace et al., 2010).

For bone resorption, the most commonly used biomarkers are divided into collagen-related or non-collagenous markers, including the hydroxylysine-glycosides, pyridinoline, deoxypyridinoline, N-terminal and C-terminal cross-linked telopeptide of type I collagen (NTX-I and CTX-I, respectively) for collagen-related proteins, and cathepsins, tartrate-resistant acid phosphatase (TRAcP), and bone sialoprotein for non-collagenous proteins in the serum (Seibel, 2005). Normally, the bone formation and bone resorption are tightly coupled to each other in the fetal development (Naylor & Eastell, 2012), therefore, the biomarkers of bone formation and resorption are useful for reflecting the properties of fetal bone development.

Maternal malnutrition is a major factor contributing to the adverse pregnancy outcomes for human beings and livestock. Some previous researches had reported that maternal malnutritional intake during middle- or late-gestation can also negatively impact fetal growth trajectory, and result in intrauterine growth retardation (IUGR) (Sharma, Shastri & Sharma, 2016), fetal growth restriction (Malhotra et al., 2019), low birth weight (Colón-Ramos et al., 2015), high cardiovascular disease (Zohdi et al., 2014), and fetal kidney development issue (Wood-Bradley et al., 2015). MNR affects fetal development by first to support the brain, heart and liver development by limiting bone development, further results in long-term consequences on postnatal health of offspring (Zhu et al., 2006). For adults, the influences of dietary nutrients on mesenchymal stem cells, including osteoblasts and osteoclasts, are complex, some nutrients, like calcium, magnesium, silicon, vitamin D/K/A/C/B, protein, iodine, docosahexaenoic acid, phosphorus, potassium and boron, can promote bone formation, whereas others (excessive zinc, manganese, copper) may have adverse effects on bone formation (Price, Langford & Liporace, 2012).

Bone is identified as an endocrine organ regulating glucose and energy metabolism (Lee et al., 2007). The lacking of osteoblast-secreted osteocalcin in mice decreases β-cell proliferation, glucose intolerance and insulin resistance, further proving that skeleton has been accompanying with an endocrine regulation in sugar homeostasis (Lee et al., 2007). The maternal nutrition status is the key factor for fetal bone development. For instance, the insufficiency of maternal vitamin D is associated with fetal health issue in later life, childhood rickets, schizophrenia and type 1 diabetes (Mahon et al., 2010). Vitamin D insufficiency can also influence the fetal femoral development (Wheeler et al., 2018).

Meanwhile, it has been demonstrated that a short-term maternal energy restriction in late-gestation of beef calves influences gene expression related to energy metabolism, immunity, stress response and muscle contraction (Sanglard et al., 2018). Impacts of prenatal nutrition on ruminant production and performance have also been investigated: a focus on growth and metabolic and endocrine function in sheep (Khanal & Nielsen, 2017). Therefore, it would be essential to depict the fetal bone development profiles with the maternal bone metabolic markers, to monitor fetal bone development performances with maternal bone metabolic proteins.

Machine learning (ML) is a kind of artificial intelligence with the statistical methods for clinical medicine data classification. Until right now, several ML techniques have been widely applied in clinical bone metabolism disease or bone researches with higher accuracy performance for diagnosis of osteopathy. In here, we briefly summarize a few successful applications of machine learning on osteopathy, such as the osteoporosis risk assessment for postmenopausal women (Yoo et al., 2013), pediatric bone age assessment (Halabi et al., 2019), the occurrence of bisphosphonate-related osteonecrosis (Kim et al., 2018), bone surface modifications (Dominguez-Rodrigo, 2019), trabecular bone mechanics (Sohail et al., 2019), or bone marrow associated with relapsed acute leukemia (Li et al., 2019a; Li et al., 2019b). However, none of them reported the association of fetal bone development with maternal bone metabolites by machine learning techniques.

Herein, we searched for the best classification models that will be able to predict the bone parameters (femur and humerus weight, diameter and length) of the fetus by using Machine Learning methods with goat serum metabolic data and experimental conditions as inputs. Previous works demonstrated the efficiency of mixing original inputs with the experimental conditions into experiment-centered features (Moving Averages, MAs) (Liu et al., 2017a; Liu et al., 2017b; Ran et al., 2016). We compared this methodology with the classical machine learning (ML) where the experimental condition information is included as input features using one-hot representation (Pedregosa et al., 2011). The power of ML algorithms had been successfully demonstrated in many biomedical applications (Le & Ou, 2016; Le, 2019; Le et al., 2019).

## Materials & Methods

### Experimental design and animal management

All the protocols for use of animal and experimental procedure in present study were approved by the Animal Care Committee according to the Animal Care and the Use Guidelines of the Institute of Subtropical Agriculture, Chinese Academy of Sciences (ISA-CAS), Changsha, China (No. KYNEAAM-2015-0009).

The experiment was conducted to discover the influence of MNR on the fetal bone growth performance in the middle (from days 45 to 100) and late gestation (from days 96 to 135) periods. Forty healthy female goats (*Liuyang* black goat, a Chinese local meat-production goat breed), in 2nd parity with the similar initial body weight and genetic background were collected and assigned to four groups (10 maternal goats for each) in a completely equally randomized design. These groups included the control (C) group in middle gestation (C-M), maternal nutrition restriction (R) group in middle gestation (R-M), C group in late gestation (C-L) and R group in late gestation (R-L), respectively.

The goats in control group were fed with 100% maintenance requirements of meat-producing goats of China (MpGC-2004), where, R group with 60% maintenance requirements of MpGC-2004 (Zhou et al., 2019). All experimental goats were provided by *Liuyang* Black Goats Nutritional Metabolism Innovation Breeding Base of ISA-CAS, natural crossing with breeding male goats after synchronization. The mating day was recorded to calculate the gestation day. All maternal goats used were free-range grazing before the experiments of maternal nutritional restriction. In the trial period, each pregnancy goat was kept in a well-ventilated individual cage with the proper temperature and humidity with free access to clean drinking water in an adaption of 3 days.

The average daily intake was measured and recorded for each pregnant goat. The ratio of concentrate to roughage (fresh crushed *Miscanthus spp.*) fed was set as 4:6 for middle gestation, and 6:4 for late gestation. The ingredients of feed concentrate on a dry matter (DM) basis are shown in Table 1. In general, the amount of intake increased gradually with increasing gestational days. The initial daily intake of pregnancy goats was around 0.85–0.95 kg, 1.15–1.25 kg for middle and late gestation periods, respectively. The feed amount of R group was adjusted weekly to 60% average daily intake of C group in the previous week. Each goat was fed twice daily at 08:30 h and 17:00 h. Daily intake of concentrate and forage was recorded.

**Table 1:** Ingredients and compositions of the experimental concentrate. Premix of Mineral and Vitamin used was the same with one of our previous work.

**Ingredients**	**DM basis, %**	**Chemical compositions**	**DM basis, %**
Corn	67.00	Dry matter, DM	89.7
Soybean meal	20.65	Crude protein, CP	21.94
Fat powder	8.00	Acid detergent fiber, ADF	54.00
Calcium bicarbonate	0.93	Neutral detergent fiber, NDF	35.00
Calcium carbonate	0.97	Calcium, Ca	0.68
Sodium carbonate	0.45		
Premix of Mineral and Vitamin^1^	2.00		

In the gestation days 100 and 135, maternal goats were slaughtered and used to collect bone and blood samples. During the experimental period, some pregnancy goats aborted for some uncontrolled reasons. In total, twenty-four maternal goats (nine goats for C-M group, six goats for R-M group; four goats for C-L and five goats for R-L) were slaughtered to collect bone and blood samples. In addition, 34 fetal goats were collected for sampling in different treatments and gestation periods, including 10 fetuses in C-M, 10 fetuses in R-M, six fetuses in C-L and eight fetuses in R-L, respectively.

### Sample collecting and analysis

The vein blood and umbilical cord blood samples were collected with normal vacuum blood collection tubes from jugular vein and umbilical cord before slaughter, respectively. All blood samples were allowed to stand for 2 h, centrifuged at 3,000 g/min for 15 min at 4 °C, and separated into 1.5 ml centrifugation tubes, stored at −80 °C refrigerator until further analysis. After slaughtered, the left femur and left humerus of the young and maternal goats were collected entirely and peeled off the associated soft tissue completely. The bone samples were washed with normal saline, dried with filter paper, and recorded the bone weights. The length and diameter of the middle spine of bone were measured with a Vernier Caliper and recorded all the parameters.

We used ELISA kits to determine the dynamic profiles of some skeleton ossification-associated parameters in the serum, like parathyroid hormone (PTH), bone alkaline phosphatase (BALP), osteocalcin (also known as bone gamma-carboxyglutamic acid containing protein, BGLAP), tartrate-resistant phosphatase (TRAP), type I collagen amino terminal peptide (INTP), and type I collagen carboxy terminal peptide (CTX-I) with the specific ELISA kits. Among, the PTH kit (catalog: CSB-E13082G; lot: C0351620332), BALP kit (catalog: CSB-E13080G; lot: G13035060), BGLAP kit (catalog: CSB-EL002682GO; lot: F30035061) were purchased from CUSABIO Biotech. Co., Ltd. The TRAP kit (catalog: MBS014917; lot: #0812015), INTP kit (catalog: MBS028250; lot: #0812015), and CTX-I kit (catalog: MBS267426; lot: #36271309) were made in USA, and purchased from Well Biological Science Co., Ltd.

### Database construction and modelling

#### Original experimental data resources

In present work, the fetus and maternal femur and humerus bone weights were collected and measured in the conditions of maintenance nutrient requirements (MNR) and nutrition restriction (60% MNR) for goats in the middle and late gestation periods. We also determined the serum bone metabolic proteins of young and maternal goats, including PTH, BALP, BGLAP, INTP, TRAP, and CTX-I. In total, there were 61 fetus instances for the bone parameters and 58 instances for the goat serum bone metabolic proteins. As bone parameters there are femur weight (Fw, g), femur length (Fl, mm), femur diameter (Fd, mm), humerus weight (Hw, g), humerus length (Hl, mm), and humerus diameter (Hd, mm). The values were measured in three types of experimental conditions (ECs): animal type (Animal), gestation periods (Period), and nutrient restriction (Treat). Animal included 34 foetuses and 24 maternal goats, gestation periods were about middle and late gestations, and nutrient restriction parameters were 100% or 60% nutrient requirements for pregnancy maternal goats.

**Figure 1:**
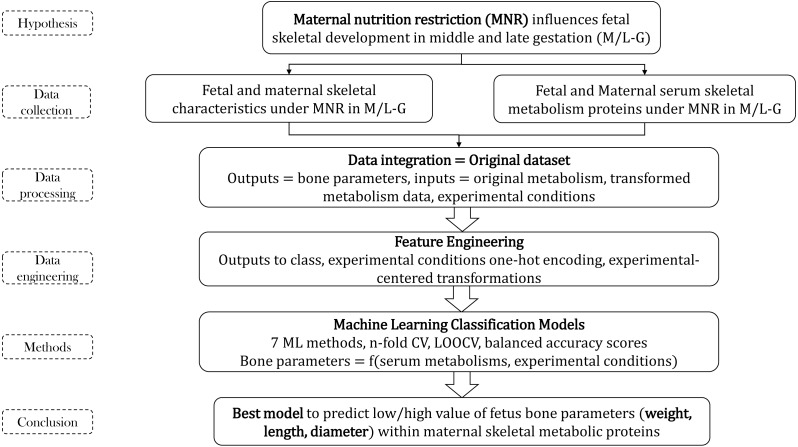
Methodology flow for bone parameters–serum metabolism study.

#### Dataset construction and predictive modelling

The entire project was structured in two principal parts: (1) dataset pre-processing before ML and (2) building of ML classifiers that are able to predict the bone parameters using serum metabolic data and experimental conditions information. Each part had several sections presented below (see Fig. 1).

The entire project calculations were included into two open repositories, GitHub and GitLab (Munteanu & Liu, 2019a; Munteanu & Liu, 2019b). At first, two scripts (Bones_1_CreateMA.ipynb, Bones_2_CreateMAi.ipynb) were integrating the data, transforming the output variables into classes, and calculated new features such as Moving Averages (MAs, experimental-center transformations = difference between original values and the average of values in specific ECs) of six bone metabolic features using 3 ECs (see Eq. (1)), and added probabilities of the separated and mixed ECs.

(1) \begin{eqnarray*}{\mathrm{MA}}_{ij}={\mathrm{Avg}}_{i}({\mathrm{Feature}}_{ij})-{\mathrm{Feature}}_{ij}\end{eqnarray*}

Feature_ij_ is the bone metabolic input feature *j* measured in experimental condition EC *i*. Avg_i_ (Feature_ij_) represents the mean of all metabolic features *j* in EC *i*. These are the script transformations of the initial data:

• Bone parameters and metabolic data were integrated using the data from serum ID and ECs: SerNum, Animal, Treat, and Period. A dataset with 56 instances was created.
• The output variables were transformed using *Robust* scaler (manage better the outliers) for each type of Animal. The continuous values of the output variables were converted into classes using a *cutoff* = 0 as the median of the values because of the *Robust* transformation. Six output variables would be used for the classifiers: Fw_Class, Fl_Class, Fd_Class, Hw_Class, Hl_Class, Hd_Class. The reason for separated transformation is that the values of the Fetus and Mon are already very different and a general scaling + cutoff will separate the values by animal type. In consequence, the models would predict if an output is from Fetus or Mon, but not if it is low or high values. Our models would be predictable that a specific output variable had low and high values.
• Feature engineering for inputs using experimental conditions and bone metabolic proteins: MAs for 6 metabolic variables (Metab: PTH, BALP, BGLAP, INTP, TRAP, CTX-I) using 3 ECs (Animal, Treat, Period). The results were MA_mix_ = MA for a set of ECs (6 features = 6 metabolic variables * 1 set of ECs): MA-PTH-experim, MA-BALP-experim, MA-BGLAP-experim, MA-INTP-experim, MA-TRAP-experim, MA-CTX-I-experim. In addition, MAs for each type pf EC were calculated (18 new features = 6 metabolic variables * 3 ECs): MA-PTH-Animal, MA-BALP-Animal, MA-BGLAP-Animal, MA-INTP-Animal, MA-TRAP-Animal, MA-CTX-I-Animal, MA-PTH-Treat, MA-BALP-Treat, MA-BGLAP-Treat, MA-INTP-Treat, MA-TRAP-Treat, MA-CTX-I-Treat, MA-PTH-Period, MA-BALP-Period, MA-BGLAP-Period, MA-INTP-Period, MA-TRAP-Period, and MA-CTX-I-Period.
• One-hot encoding of ECs to be used as inputs in classical ML: one column for each value of the ECs (6 new features = 3 ECs * 2 values): Animal_Fetus, Animal_Mon, Treat_Con, Treat_Res, Period_Late, Period_Mid;
• Added probabilities for each ECs in the dataset (P_i_ = 3 new features for each of 3 ECs): Prob_Animal, Prob_Treat, Prob_Period;
• Added probability for mixed ECs in the dataset (1 new feature for 1 set of ECs): Prob_Mix (P_mix_).

In the next script (Bones_3_CreateDatasets.ipynb), different types of data were used to generate 72 datasets in order to check what data were needed for the best classification model that can predict bone parameters using metabolic data: six output variable classes, six one-hot encoding of ECs, six metabolism, six mixed MAs of metabolic data, 18 MAs of metabolic data for each ECs, one mixed probability, and three EC probabilities.

With these 72 datasets we tested seven ML classifiers with default parameters from sklearn/python (504 prediction models): KNN (KNeighborsClassifier), SVM linear (Support Vector Classifier, SVC, with linear kernels, SVM, Support Vector Machines), SVM (SVC with RBF kernels), LR (LogisticRegression), DT (DecisionTreeClassifier), RF (RandomForestClassifier, RF, Random Forest), and XGB (XGBClassifier, XGB, XGBoost). The used pipelines consisted in (1) scaling input features with RobustScaler and the classifier. Two types of cross-validation were tested: n-fold CV (*n* = 2, 3, 5, and 10) and LOOCV (Leave-one-out cross-validation). The best CV for a small dataset is always LOOCV. The results for n-fold CV are presented only in the project repository. Balanced accuracy was used as optimization score for ML methods in the case of n-fold CV (Bones_4_OuterCV-Pipelines-ACC.ipynb) and LOOCV (Bones_4_OuterCV-Pipelines-LOOCV.ipynb). The scripts tested both the MA datasets and the classical ML datasets using one-hot encoding of ECs (Bones_4_OuterCV-Pipelines-LOOCV-OneHot.ipynb). Therefore, we tested if MAs inputs were better than the original metabolic inputs with one-hot encoding ECs. In other words, we tested what methodology was better for our task: the encoding of ECs into MAs (mixed information of metabolism and ECs) or the one-hot representation of ECs as input binary variables (1/0 values). In ML, the categorical features cannot be handled by all the ML methods. Therefore, there is a need for the transformation of the categorical values into numerical ones. The one-hot encoding is used when there is no natural order between the categories. If we use the simple integer encoding (an integer for any category), we will allow the model to assume a natural ordering between categories. This could produce poor performance or unexpected results.

In addition, we evaluated the feature importance by feature removal method for each of the best models. Thus, for each linear or nonlinear SVM model, we removed each input variable and calculated the new ACC values (LOOCV). (see Bones_5_FeatureI-mportance-Pipelines-LOOCV.ipynb). The differences between the ACC with pool dataset (all features) and the new ACC for each dataset without one feature were presented. If the removal of a feature will determine the decrease of the ACC, this feature is considered important. If the removal of a feature will not change the ACC, the feature is not important for the model. If the removal of one feature will increase the ACC, this feature represents a noise for the model and it should be eliminated. For the best models for each output, a different feature selection was tested: create models with the same ML method and dataset but using only one feature (Bones_6-OneFeature-Models-LOOCV-ACC.ipynb).

## Results

### Fetal bone developmental profiles

The effects of MNR on gestation goats for fetal bone growth profiles (including weight, length and diameter of femur and humerus) are presented in Table 2. In the middle gestation, MNR improved the fetal bone weight, but decreased the length and diameter of bone (femur and humerus). In the late gestation, MNR enhanced the weight and diameter of humerus and femur, and humerus length, but decreased the femur length. However, for the local goat nutritional research, except the nutritional status providing the nutritional requirements of fetal skeletal development, the genetic factors of each individual might also play an important role in fetal skeletal development.

**Table 2:** Effect of intake restriction on fetal bone properties in middle- and late- gestations of goats.

**Item**	**Index**	**Middle gestation (100 d)**				**Late gestation (135 d)**			
		C	R	SE*m*	*P* value	C	R	SE*m*	*P* value
Femur	weights (g)	9.97	10.84	0.625	0.33	79.93	86.02	6.102	0.50
	Length (mm)	54.9	53.1	1.50	0.39	139.6	124.8	26.70	0.70
	Diameter (mm)	1.5	1.3	0.06	0.02	16.8	17.2	0.84	0.77
Humerus	weights (g)	9.87	10.45	0.584	0.49	78.85	79.76	9.796	0.95
	Length (mm)	54.1	53.6	1.62	0.85	126.3	130.5	5.72	0.62
	Diameter (mm)	132.2	133.9	4.04	0.76	16.3	16.8	1.22	0.77

**Notes.**

C represents the control group feeding with 100% feeds; R represents restriction group feed with 40% of feeds, BMD, Bone mineral density (g/cm^3^), SEm, standard error of measurement.

### Nutritional restriction on goat bone metabolic indexes

Except the nutritional supplementation, the regulators or biomarkers of bone metabolism were also taken into consideration for fetal bone development. Here, we measured the goat bone metabolic proteins (PTH, BALP, BGLAP, TRAP, INTP and CTX-1) for maternal jugular venous serum and fetal umbilical cord serum (showed in Table 3 and Table 4, respectively). For maternal goats, the nutritional factor, MNR, reduced the levels of PTH, BALP, BGLAP, TRAP, INTP and CTX-1 in peripheral serum.

**Table 3:** Effects of maternal intake restriction on bone turnover metabolic proteins of maternal peripheral blood in middle- and late- gestations of goats.

**Item**	**Middle gestation (100d)**				**Late gestation (135d)**			
	**C**	**R**	SE*m*	*P* value	**C**	**R**	SE*m*	*P* value
PTH	55.67	45.51	8.23	0.354	68.50	67.80	5.240	0.924
BALP	226.11	123.99	55.84	0.19	69.20	119.50	33.160	0.343
BGLAP	22.84	16.39	9.510	0.605	11.60	10.60	0.990	0.537
TRAP	63.25	61.17	6.700	0.814	30.00	29.50	2.800	0.918
INTP	2.24	1.34	0.714	0.358	0.22	0.72	0.121	0.025
CTX-I	7.21	6.52	0.355	0.206	1.10	1.15	0.054	0.532

**Notes.**

C represents Control group, R represents 40% maternal nutrition restriction group; SEm = standard error of measure, in addition, Parathyroid hormone (PTH, ng/mL), Bone Alkaline Phosphatase (BALP, mU/mL), Osteocalcin also known as bone gamma-carboxyglutamic acid-containing protein (BGLAP, ng/mL), Tartrate-resistant Phosphatase (TRAP, U/L), Cross-linked N-terminal telopeptides of type 1 collagen or named Intact N- terminal propeptide of Type 1 procollagen (NTx, INTP, ng/mL), Cross-linked C-terminal telopeptides of type I collagen (CTx, CTX-I, ng/mL), respectively.

**Table 4:** Effects of maternal intake restriction on fetal bone metabolic proteins of placental blood in middle- and late- gestations of goats.

Item	**Middle restriction**				**Late restriction**			
	C	R	SE*m*	*P* value	C	R	SE*m*	*P* value
PTH	900.13	1000.47	78.81	0.39	768.20	647.80	121.740	0.44
BALP	3.215	3.441	0.425	0.67	3.14	3.24	0.715	0.90
BGLAP	4.27	4.38	0.102	0.37	4.43	4.06	0.249	0.26
TRAP	58.30	61.97	6.56	0.67	38.80	39.60	1.880	0.75
NTx	0.59	0.57	0.040	0.64	0.83	0.46	0.091	<0.01
CTx	1.23	0.94	0.092	0.03	4.29	3.74	0.33	0.23

**Notes.**

C represents Control group, R represents 40% maternal nutrition restriction group; SEm = standard error of measure, in addition, Parathyroid hormone (PTH, ng/mL), Bone Alkaline Phosphatase (BALP, mU/mL), Osteocalcin also known as bone gamma-carboxyglutamic acid-containing protein (BGLAP, ng/mL), Tartrate-resistant Phosphatase (TRAP, U/L), Cross-linked N-terminal telopeptides of type 1

Under the malnutritional condition, low maternal BGLAP level represents low efficient maternal bone turnover rate. However, our results showed that the other biochemical markers of bone turnover, TRAcP, had no difference in malnutrition condition. Compared with significant differences in bone metabolic proteins for maternal individual, fetal bone metabolic proteins showed a minor variation in the control and malnutrition groups. The NTx and CTx were reduced in response to maternal malnutrition, CTx was obviously lower in the middle-gestation, while NTx significantly lower in late-gestation. The PTH for maternal malnutrition was higher or lower in middle- and late- gestations, respectively. Whereas, the fetal BALP, BGLAP, and TRAP showed no statistical difference between the control and malnutritional groups.

### Machine learning predictive models

Seventy-two datasets were used to find the best ML model to predict six bone parameters by using seven ML methods such as KNN, SVM linear (linear kernels), SVM (RBF kernels), LR, DT, RF and XGB. The input features came from 6 one-hot encoding of ECs, six metabolic original data, six mixed MAs of metabolic data, 18 MAs of metabolic data for each ECs, 1 mixed probability, and three EC probabilities. The Python script was used Robust scaler for data scaling, cross-validation (n-fold CV, where *n* = 2, 3, 5, 10 and LOOCV), and balanced accuracy as performance score.

Table 5 presents the best accuracy (ACC) values obtained for all outputs using only LOOCV. For 5-, 10-fold CV results or other details, see the repository in GitLab (Munteanu & Liu, 2019a) or GitHub (Munteanu & Liu, 2019b).

The best CV method for a small dataset was the LOOCV one. The results based on this CV showed models with all ACC >0.70, with classifiers of five from six outputs with ACC between 0.75 and 0.90, and one classifier with ACC >0.90. In this study, we observed the performance of SVM method with bone dataset and LOOCV (all classifiers are SVM/SVM linear). The most preferred inputs were MAmix, and Metab. MAi was used only for one output and probabilities are missing. The best classifier with ACC of 0.911 was obtained for prediction of Fw by using SVM with RBF non-linear kernels and three types of inputs such as MAi, MAmix, and Metab. There were three outputs (Fl, Hw and Hl) where the best models used only six MAmix features (MAs for the set of ECs: MA-PTH-experim, MA-BALP-experim, MA-BGLAP-experim, MA-INTP-experim, MA-TRAP-experim, and MA-CTX-I-experim). The model for Fl prediction could achieve an ACC of 0.875 with only 6 input features (MAmix) and a SVM model with linear kernels (equivalent of a linear model). Fd prediction model increased a little with the ACC of 0.893 by adding to MAmix the Metab features (6 original metabolic features) and by using SVM with RBF non-linear kernels. Hw could be predicted with less ACC of 0.75, with only MAmix inputs and SVM with linear kernels. The model for Hd prediction had a good ACC of 0.857 using SVM with RBF kernels and MAmix + Metab inputs. An interesting pattern was observed for the preference of input information for each type of bone parameter: MAmix was used of bone length (Fl, Hl) prediction, and MAmix + Metab for bone diameter (Fd, Hd) prediction. In the case of bone weight (Fw, Hw), the preference of inputs was different.

**Figure 2:**
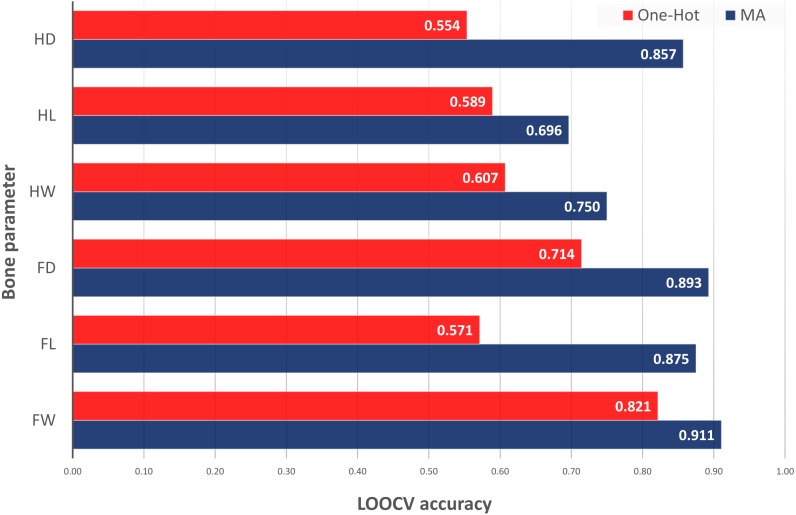
LOOCV accuracy of classification models for bone parameter prediction using one-hot vs. MA encoding of experimental conditions into the input features (direct vs. mixed with metabolic data).

The last test was the comparison of the best ACC values obtained by different inclusion of EC information into the model inputs: classical one-hot encoding of ECs as binary inputs for each categorical value versus MA encoding as difference between the original metabolic data and the average of this data in specific ECs. In Fig. 2, it could be observed that the classical use of categorical EC values and original metabolic features was unable to provide good results with the current small dataset for bone parameters prediction. Thus, one-hot encoding of ECs was able to classify only Fd with ACC of 0.714 (versus 0.893 with MAs) and Fw with ACC of 0.82 (versus 0.911 with MAs). One-hot encoding models were not able to predict Hd, Hl and Fl (ACC < 0.60). This test demonstrated the need of MAs to be able to classify low and high values of bone parameters. Additional details about the one-hot encoding results can be found in our repository notebooks (Munteanu & Liu, 2019a).

### Feature importance

To evaluate the feature importance for the best model for each output, we presented a feature selection method by individual feature removal (see Bones_5_Feature-Importance-Pipelines-LOOCV.ipynb). The more an attribute is applied for making key decisions with decision trees, the higher its relative importance (Tang, Alelyani & Liu, 2014). This importance is calculated explicitly for each attribute in the dataset, allowing attributes to be ranked and compared to each other. The ranking of features was based on the difference between the ACC calculated without a feature (new-ACC) and original ACC calculated with all features (see Table 6). If the difference is smaller (more negative), it means that, if you remove that specific feature, the ACC of the model will suffer more (feature is more important). If the ACC difference is positive, it means that, if you remove that specific feature, you will obtain a better ACC (feature not important or even noise for the model). All details are available in our GitLub repository (FeatImpbyRemoval-LOOCV_SVMlinear_ACC.csv, FeatImpbyRemoval-LOOCV_ACC.csv).

**Table 5:** Best bone parameters prediction accuracy for LOOCV.

**Output**	**ML method**	**Input features**	**ACC**
Fw	SVM	MAi, MAmix, Metab	**0.911**
Fl	SVM linear	MAmix	0.875
Fd	SVM	MAmix, Metab	0.893
Hw	SVM linear	MAmix	0.750
Hl	SVM	MAmix	0.696
Hd	SVM	MAmix, Metab	0.857

**Table 6:** Feature importance for the best models.

**Output**	**Removed feature**	**New ACC without a feature**	**Difference with Pool ACC**	**Output**	**Removed feature**	**New ACCwithout** a feature	**Difference with Pool ACC**
Fw	MA-PTH-Animal	0.857	−0.054	Hw	MA-CTX-I-experim	0.714	−0.036
	MA-BALP-experim	0.875	−0.036		MA-BGLAP-experim	0.732	−0.018
	MA-BALP-Animal, MA-BGLAP-Animal, MA-INTP-Animal, MA-TRAP-Animal, MA-PTH-Treat, MA-BALP-Treat, MA-BGLAP-Treat, MA-TRAP-Treat, MA-PTH-Period, MA-BALP-Period, MA-BGLAP-Period, MA-BGLAP-experim, MA-INTP-experim, MA-CTX-I-experim, PTH, BALP, BGLAP, INTP, TRAP	0.893	−0.018		MA-INTP-experim	0.732	−0.018
	MA-PTH-experim	0.946	0.035		MA-TRAP-experim	0.732	−0.018
Fd	MA-INTP-experim	0.661	−0.232		MA-BALP-experim	0.750	0.000
	MA-PTH-experim	0.679	−0.214		MA-PTH-experim	0.875	0.125
	MA-CTX-I-experim	0.714	−0.179	Hd	MA-PTH-experim	0.714	−0.143
	MA-BALP-experim	0.768	−0.125		MA-BALP-experim	0.732	−0.125
	PTH	0.804	−0.089		MA-BGLAP-experim, MA-TRAP-experim	0.804	−0.053
	MA-TRAP-experim, BALP, BGLAP, INTP	0.821	−0.072		PTH, BALP, BGLAP	0.821	−0.036
	MA-BGLAP-experim, CTX-I	0.857	−0.036		MA-INTP-experim, INTP, CTX-I	0.839	−0.018
	TRAP	0.875	−0.018		MA-CTX-I-experim	0.857	0.000
Fl	MA-CTX-I-experim	0.696	−0.179		TRAP	0.875	0.018
	MA-BALP-experim	0.821	−0.054	Hl	MA-PTH-experim, MA-BALP-experim	0.643	−0.053
	MA-INTP-experim	0.821	−0.054		MA-CTX-I-experim	0.661	−0.035
	MA-TRAP-experim	0.839	−0.036		MA-INTP-experim, MA-TRAP-experim	0.679	−0.017
	MA-BGLAP-experim	0.857	−0.018		MA-BGLAP-experim	0.696	0.000
	MA-PTH-experim	0.911	0.036				

 In the case of Fw best model (SVM RBF, ACC = 0.911), the most important features were MA-PTH-Animal and MA-BALP-experim with a decrease of ACC of 0.054 and 0.036. In addition, MA-PTH-experim represents a noise for the model and its removal increased the ACC of the model with 0.035. Thus, the best model for Fw could be considered the one obtained without MA-PTH-experim, with test ACC of 0.946. Therefore, PTH moving average for each type of animal was important for the model but not the PTH moving average in mixed ECs. The feature importance for Fd best model (SVM RBF, ACC = 0.893) was different. The impact of feature removal of moving averages for INTP, PTH, CTX-I, and BALP in mixed ECs was very important, with differences in ACC between 0.125 and 0.232. The original metabolic features were less important. Fl best model (SVM linear, ACC = 0.875) preferred MA-CTX-I-experim and the ACC was improved with 0.036 by removing MA-PTH-experim. Therefore, the best model for Fl became the one without MA-PTH-experim, with a test ACC of 0.911.

The Hw best model (SVM linear, ACC = 0.750) showed preference for MA-CTX-I-experim and considered MA-PTH-experim as noise. By removal of this feature the model ACC was improved with 0.125. Thus, the new ACC of 0.875 represented the best performance for Hw. In contrast, Hd best model (SVM, ACC = 0.857) preferred MA-PTH-experim and MA-BALP-experim, the removal of MA-CTX-I-experim did not change the ACC, but the elimination of TRAP could increase the model performance with 0.018. Thus, the best model for Hd became the one without TRAP, with ACC of 0.875. Hl best model (SVM RBF, ACC = 0.696) preferred the same MA-PTH-experim and MA-BALP-experim, but it could remove MA-BGLAP-experim without any change of ACC.

As a general view for the feature importance, it is noticeable that the moving averages in mixed ECs were generally more important for the majority of the models. MA-PTH-experim was important for Fd, Hd, and Hl but its removal for Fw, Fl and Hw improved the model performance. In general, we observed the important role of PTH and BALP mixed with ECs.

With the best models and ML methods obtained Table 5, one feature models have been tested for all outputs. Therefore, Table 7 presents the ACC values for these models and the differences with the pool feature models. In the case of the ACC improvements, the text has a bold style. First of all, it should be pointed out that the current study is interested into models that include experimental conditions in order to use them to understand the relations with the fetus bones.

**Table 7:** Accuracies of the best models using only one feature.

**Output**	**Feature**	**ACC**	**Diff. with Pool ACC**	**Output**	**Feature**	**ACC**	**Diff. with Pool ACC**
Fd	CTX-I	0.411	−0.482	Hd	CTX-I	0.250	−0.607
	MA-CTX-I-experim	0.429	−0.464		MA-CTX-I-experim	0.429	−0.428
	MA-INTP-experim	0.554	−0.339		TRAP	0.607	−0.250
	TRAP	0.589	−0.304		MA-PTH-experim	0.625	−0.232
	MA-PTH-experim	0.607	−0.286		MA-INTP-experim	0.732	−0.125
	MA-BALP-experim	0.714	−0.179		BALP	0.821	−0.036
	INTP	0.768	−0.125		INTP	0.857	0.000
	MA-BGLAP-experim	0.786	−0.107		**BGLAP**	**0.875**	**0.018**
	MA-TRAP-experim	0.786	−0.107		**MA-BGLAP-experim**	**0.911**	**0.054**
	BALP	0.839	−0.054		**MA-TRAP-experim**	**0.929**	**0.072**
	**BGLAP**	**0.946**	**0.053**		**MA-BALP-experim**	**0.946**	**0.089**
	**PTH**	**0.982**	**0.089**		**PTH**	**0.982**	**0.125**
Fw	MA-INTP-experim	0.714	−0.197	Hl	MA-PTH-experim	0.357	−0.339
	MA-INTP-Animal	0.768	−0.143		MA-CTX-I-experim	0.411	−0.285
	INTP	0.786	−0.125		MA-BALP-experim	0.464	−0.232
	MA-PTH-Animal	0.821	−0.090		MA-BGLAP-experim	0.500	−0.196
	CTX-I	0.821	−0.090		MA-INTP-experim	0.625	−0.071
	MA-CTX-I-Animal	0.839	−0.072		MA-TRAP-experim	0.696	0.000
	MA-CTX-I-Period	0.857	−0.054	Fl	**MA-BGLAP-experim**	**0.911**	0.036
	MA-BGLAP-Animal	0.875	−0.036		**MA-CTX-I-experim**	**0.982**	0.107
	MA-CTX-I-Treat	0.875	−0.036		**MA-PTH-experim**	**1.000**	0.125
	MA-PTH-experim	0.893	−0.018		**MA-BALP-experim**	**1.000**	0.125
	BALP	0.911	0.000		**MA-INTP-experim**	**1.000**	0.125
	BGLAP	0.911	0.000		**MA-TRAP-experim**	**1.000**	0.125
	**MA-BALP-Period**	**0.929**	0.018	Hw	MA-BGLAP-experim	0.464	−0.286
	**MA-BALP-Animal**	**0.946**	0.035		MA-BALP-experim	0.589	−0.161
	**MA-BALP-Treat**	**0.946**	0.035		**MA-PTH-experim**	**0.946**	0.196
	**MA-INTP-Treat**	**0.964**	0.053		**MA-TRAP-experim**	**0.946**	0.196
	**MA-BGLAP-experim**	**0.964**	0.053		**MA-CTX-I-experim**	**0.982**	0.232
	**MA-CTX-I-experim**	**0.964**	0.053		**MA-INTP-experim**	**1.000**	0.250
	**MA-BALP-experim**	**0.982**	0.071				
	**MA-TRAP-Animal**	**1.000**	0.089				
	**MA-PTH-Treat**	**1.000**	0.089				
	**MA-BGLAP-Treat**	**1.000**	0.089				
	**MA-TRAP-Treat**	**1.000**	0.089				
	**MA-PTH-Period**	**1.000**	0.089				
	**MA-BGLAP-Period**	**1.000**	0.089				
	**MA-INTP-Period**	**1.000**	0.089				
	**MA-TRAP-Period**	**1.000**	0.089				
	**MA-TRAP-experim**	**1.000**	0.089				
	**PTH**	**1.000**	0.089				
	**TRAP**	**1.000**	0.089				

**Notes.**

ACC values corresponds to LOOCV for models the ML methods of the best models (see Table 5).

Fw models based on only one feature can be more accurate than the best model presented with the pool dataset. Thus, a better model with ACC values of 1.00 (LOOCV) could be obtained with PTH and TRAP or the mixed feature MA-TRAP-experim. A number of 18 features could be use separately to improve the best models with pool features. INTP and the mixed features with INTP are not able to generate any stable model. In consequence, the best model for Fw (ACC = 1.00) become the one that uses SVM (radial kernel) and MA-TRAP-experim.

In the case of Fd classifications, it can be observed that using simple features without experimental conditions such as BGLAP and PTH can be used as unique feature of prediction models with better ACC (maximum of 0.982). In contrast, other original features such as CTX-I, TRAP, INTP and BALP or the mixed features are not able to create better predictions. In the case of Fl, all the experimental features are improving the ACC to a maximum value of 1.00 for MA-PTH-experim, MA-BALP-experim, MA-INTP-experim, and MA-TRAP-experim.

Hd classifications could be improved if PTH, BGLAP, MA-BGLAP-experim, MA-TRAP-experim and MA-BALP-experim are used as single feature with SVM. The best improvements were obtained with PTH (ACC = 0.982) and MA-BGLAP-experim (ACC = 0.946). Hl classifications cannot be improved be using one feature. Hw can be improved to ACC = 1.00 by using only MA-INTP-experim. It implied that the maternal plasma metabolite (INTP) can be used to perfectly match or predict the fetal humerus growth performance (high or low weight) in the experimental conditions.

## Discussion

### Nutritional restriction on bone metabolic profiles

Maternal limited nutrition intake during gestation impairs fetal growth and development by exacerbating deleterious outcomes (Abu-Saad & Fraser, 2010; Wu et al., 2004; Wu, Imhoff-Kunsch & Girard, 2012), such as intrauterine growth restriction (IUGR) (Sharma, Shastri & Sharma, 2016), birth defects and low birth weight (Abu-Saad & Fraser, 2010), bone development (Kueper et al., 2015), and low fetal immunity (Macpherson, De Aguero & Ganal-Vonarburg, 2017). Generally speaking, maternal instinct is to mobilize the body potential with the priority to meet the nutritional requirements of the fetal or placental growth in malnutrition condition (Wu, Imhoff-Kunsch & Girard, 2012). In present work, the biomarkers of bone resorption (BGLAP, TRAP, and CTX-1) or bone formation (BALP, and INTP) in serum reduced in goat group under nutrient restriction, indicating maternal malnutrition indeed influences the maternal or fetal bone metabolic turnover (Kovacs, 2001; Wu et al., 2004; Wu, Imhoff-Kunsch & Girard, 2012). According to the previous report, fetal growth and development is most vulnerable in the early-gestation (Wu et al., 2006). However, the influence of maternal nutrition intake also plays the vital role for fetal bone development in middle- and late- gestations. Under the malnutritional condition, low maternal osteocalcin (BGLAP) level represents low efficient maternal bone turnover, which may be decreased during pregnancy (Charles et al., 1985; Naylor et al., 2000).

The results also showed that fetal bone metabolites showed a minor discrepancy compared to high difference of mother in malnutritional condition. The BALP, a bone-specific isoform of ALP on the surface of osteoblasts, an important biomarker reflects the biosynthetic activity of bone-forming cells (Shipman, Holt & Gama, 2013; Tanaka et al., 1997). In addition, BGLAP level in the circulation system is also a symbol for evaluating the bone formation (Booth et al., 2012; Mendes et al., 2019). The serum BALP level decreased under the nutrition restriction, reflecting that malnutrition (protein deficiency, zinc deficiency or malnutrition) negatively regulates the secretion of maternal bone alkaline phosphatase (Sun et al., 2011). Maternal malnutrition might decrease the glucose metabolic process by decreasing osteocalcin level in maternal peripheral blood by regulating the insulin secretion and sensitivity (Ferron & Lacombe, 2014; Kanazawa et al., 2009) in middle-gestation.

In adults, the increasing TRAcP expression might be associated with the osteoporosis (Solberg et al., 2014), cortical bone mineral content and density (Gradin et al., 2012). Interestingly, in present work, we found that the biomarkers of bone turnover, the bone metabolic markers, including NTx, CTx, BALP, BGLAP and TRAP except PTH, decreased along with gestation time (from middle-gestation to late-gestation). The BGLAP concentration increased in maternal serum compared to that of fetus, reflecting enhanced osteoblastic activity. The bone turnover metabolic proteins decreased in late-gestation is due to maternal goats giving the priority to meet the nutritional requirements of fetal skeletal development through decreasing the self- bone turnover or resorption. The mother limits self–bone turnover/resorption rate or further to release more mineral contents of maternal bones to sustain the fetal bone development while undernutrition issues.

### Machine learning predictive models for bone metabolic profiles

Machine learning (ML) is a kind of artificial intelligence with the statistical methods for clinical medicine data classification. Until right now, several ML techniques have been widely applied in clinical bone metabolism disease or bone researches with higher accuracy performance for diagnosis of osteopathy. In here, we briefly summarize a few successful applications of machine learning on osteopathy, such as the osteoporosis risk assessment for postmenopausal women (Yoo et al., 2013), pediatric bone age assessment (Halabi et al., 2019), the occurrence of bisphosphonate-related osteonecrosis (Kim et al., 2018), bone surface modifications (Dominguez-Rodrigo, 2019), trabecular bone mechanics (Sohail et al., 2019), or bone marrow associated with relapsed acute leukemia (Li et al., 2019a; Li et al., 2019b). For instance, the variability of sclerostin (a physiological inhibitor of bone formation) varied with sex, 25-OH-D and phosphorus levels in haemodialysis (HD) patients by multivariate regression analysis (Pietrzyk et al., 2019), low serum parathyroid hormone associated with malnutrition-inflammation complex in chronic kidney disease by Chis-square test, linear regression and multivariate logistic regression analysis (Dukkipati et al., 2010). In addition, the abnormalities of biochemical markers of bone turnover, ALP and PTH, are associated with mortality of HD patient across age by Cox proportional hazard models (Lertdumrongluk et al., 2013), and a decrease in intact parathyroid hormone (iPTH) is associated with higher mortality (Villa-Bellosta et al., 2017). There are also some previous works that combined with the machine learning algorithms with data processing analysis of moving average and feature selection to biological phenomena (Deng et al., 2017; Liu et al., 2016a; Liu et al., 2016b). However, none of them reported the association of fetal bone development with maternal bone metabolites by machine learning techniques.

In present work, seven ML methods, including KNN, SVM linear (linear kernels), SVM (RBF kernels), LR, DT, RF and XGB, were used to find the best ML model to predict 6 bone parameters. Then, the feature selection method within individual feature removal was used to evaluate the feature importance for each output (predictive parameter). To some extent, we got some good performance predictive models for matching bone profiles with serum bone metabolic biomarkers. Generally, it can be observed that isolated PTH and its mixed features are able to improve the prediction of bone dimensions. The next important features are BGLAP and its mixed features. Therefore, it could be concluded that some original features such as PTH and BGLAP are the most important for fetal bone development performance predictions and experimental-mixed features could be used to predict bone features in specific experimental conditions. With other words, the original features could predict bone properties but the experimental conditions could offer more details.

## Conclusions

The maternal and fetal bone metabolic proteins decrease from middle to late gestation. Maternal nutrition restriction alters the bone development of offspring (bone weight, length and diameters of femur and humerus) and the fetal bone metabolic protein levels, including CTx level in middle-gestation and NTx level in late-gestion. Maternal nutrition restriction also influences the maternal bone metabolic protein compositions, resulting in lower BALP level in middle-gestation and higher BALP level in late-gestation. Furthermore, we constructed a machine learning model to elucidate the fetal bone performances associated with maternal or placental serum bone metabolic proteins. This study built the classifiers with accuracy greater than 0.70 for all bone parameters. In the view of the feature importance, the moving averages in mixed with experimental conditions are generally more important for the majority of the models. Particularly, the moving average of PTH within experimental condition (MA-PTH-experim) is important for Fd, Hd, and Hl but its removal for Fw, Fl and Hw improves the model performance. In general, we observe the important role of PTH and BALP mixed with experimental conditions. In addition, using only one feature with the Machine Learning methods of the best models, five of the six outputs can be improved: Fw with MA-TRAP-experim (ACC = 1.00), Fd with PTH (ACC = 0.982), Fl with MA-PTH-experim (ACC = 1.00), Hw with MA-INTP-experim (ACC = 1.00) and Hd with MA-BALP-experim (ACC = 0.946). Hl best model using mixed features has ACC of only 0.696. Thus, the best classification models with ACC = 1.00 were obtained for the prediction of femur weight and length, and humerus weight using support vector machines.

##  Supplemental Information

10.7717/peerj.7840/supp-1 Supplemental Information 1 Full dataset constructed of bone characteristics and bone metabolism protein profile

10.7717/peerj.7840/supp-2 Supplemental Information 2 Create moving average (MAs) for a set of experimental conditions (all EC) for goat bone metabolism profiles

10.7717/peerj.7840/supp-3 Supplemental Information 3 Create moving average (MAs) by using an individual experimental condition (individual EC) for goat bone metabolism profiles

10.7717/peerj.7840/supp-4 Supplemental Information 4 Create datasets by using the previous MAs (all EC) and (individual EC) and class transformed outputs for goat bone metabolism profiles

10.7717/peerj.7840/supp-5 Supplemental Information 5 Pipelines for classifiers using balanced accuracy and accuracy metrics for goat bone metabolism profiles

10.7717/peerj.7840/supp-6 Supplemental Information 6 Pipelines for classifiers using area under the curve (AUC) for goat bone metabolism profiles

10.7717/peerj.7840/supp-7 Supplemental Information 7 Pipelines for classifiers using LOOCV for goat bone metabolism profiles

10.7717/peerj.7840/supp-8 Supplemental Information 8 Pipelines for classifiers using LOOCV for one-hot ECs for goat bone metabolism profiles

10.7717/peerj.7840/supp-9 Supplemental Information 9 Pipelines with feature importance for classifiers with LOOCV for goat bone metabolism profiles

10.7717/peerj.7840/supp-10 Supplemental Information 10 One feature model for goat bone metabolism profiles by using feature selection method with the pipelines for classifiers with LOOCV

## Additional Information and Declarations

### Data Availability

The following information was supplied regarding data availability: Python code for machine learning is available in Github and GitLab repositories https://github.com/muntisa/Goat-Bones-Machine-Learning and https://gitlab.com/muntisa/goat-bones-machine-learning. The raw data is available in a Supplemental File.
